# Simplified versus standard EEG to measure the depth of sedation in mechanically ventilated ICU patients

**DOI:** 10.1186/cc13602

**Published:** 2014-03-17

**Authors:** T Suys, P Bouzat, A Rossetti, M Oddo

**Affiliations:** 1 Lausanne University Hospital, Lausanne, Switzerland

## Introduction

The accurate measure of sedation depth among mechanically ventilated ICU patients remains challenging. The Patient State Index (PSI) is a quantitative measure calculated using an algorithm derived from a simplified four-channel EEG. The aim of this study was to examine the value of the PSI to assess the level of sedation, and to verify its accuracy in comparison with the quantitative spectral analysis derived from a standard 19-channel EEG.

## Methods

Using the SEDLine four-channel simplified EEG system (Masimo Corporation, Irvine, CA, USA), which assessed depth of sedation through two frontal leads, we prospectively studied mechanically ventilated sedated ICU patients and examined whether the PSI was accurate to quantify the individual level of sedation. Pain stimuli were applied and changes in PSI were examined and compared with changes in electrical activity (% of delta power), measured by the frontal Fp2-Fp1 electrodes using a standard 19-channel EEG system (Viasys Neurocare, Madison, WI, USA). EEG recordings were performed simultaneously during 20 minutes, and the relationship between PSI and % of delta power was analyzed with the Pearson's correlation coefficient.

## Results

Ten consecutive patients (mean age 59 ± 12 years) were included. EEG recordings were performed on average 6 ± 6 days from ICU admission. Sedation consisted of propofol (seven patients), midazolam (two patients) or both (one patient). The median PSI was 54 (range 10 to 97), indicating large individual variations in PSI values. In contrast, the median delta power was 86.3% (range 55.2 to 99.4), indicating a deep sedation level. Pain stimuli were associated with a significant increase of PSI value from baseline 51 (range 16 to 96) to 68 (42 to 95) (*P *= 0.009). However, we found no correlation between the PSI and the % delta power, both at baseline *(R *= 0.32, *P *= 0.37) and after pain stimulation *(R *= 0.17, *P *= 0.63). The % delta power from frontal electrodes (Fp2-Fp1) was well correlated with that obtained from posterior electrodes (P4-Pz; *R *= 0.42, *P <*0.0001), and remained unchanged after pain stimulation, indeed confirming a deep sedation level in our patient cohort.

## Conclusion

Using standard EEG in this small cohort of mechanically ventilated ICU patients, a deep sedation level was frequently observed. Our preliminary data suggest that simplified EEG with the SedLine system is less accurate than the standard 19-channel EEG to assess the depth of sedation in the ICU setting.

